# Meta-analysis of the effects of levothyroxine therapy for subclinical hypothyroidism during pregnancy on offspring outcomes

**DOI:** 10.3389/fped.2025.1530859

**Published:** 2025-07-10

**Authors:** Zhiying You, Yayu Zhang, Sen Liu, Jia Li, Xiaofang Xu, Dan Song

**Affiliations:** ^1^ Department of Neonatology, Affiliated Hospital of Inner Mongolia Medical University, Huhhot, China; ^2^ Department of Ultrasound, The Inner Mongolia Hospital of Peking University Cancer Hospital, Hohhot, China

**Keywords:** pregnancy, subclinical hypothyroidism, SCH, L-T4, offspring, neonate, meta-analysis

## Abstract

**Objective:**

To conduct a systematic evaluation of the impact of levothyroxine (L-T4) therapy on birth outcomes in pregnancies complicated by subclinical hypothyroidism (SCH).

**Methods:**

A thorough literature review was conducted across several databases, including PubMed, Embase, Cochrane Library, Web of Science, Sinomed, Wanfang Data Knowledge Service Platform, China National Knowledge Internet (CNKI), and VIP Chinese Science and Technology Journal Database (VIP), to investigate the impact of L-T4 treatment of SCH during pregnancy on birth outcomes in offspring. Based on predefined inclusion and exclusion criteria, two researchers were appointed to extract data and assess the quality of the literature. Subsequently, meta-analysis was performed using RevMan 5.4 and Stata 14 software.

**Results:**

The study incorporated a total of thirty randomized controlled trials (RCTs) and cohort studies, encompassing four countries and regions. The sample included 18,568 pregnant women with SCH and 5,578 pregnant women undergoing L-T4 treatment. The meta-analysis indicated that L-T4 treatment for SCH during pregnancy may reduce the incidence of preterm birth (RCTs (RR = 0.56, 95% CI = 0.41–0.77), cohort studies (RR = 0.71, 95% CI = 0.51–0.99)) and low birth weight infants (LBWI) (RCTs (RR = 0.56, 95% CI = 0.35–0.89), cohort studies (RR = 0.71, 95% CI = 0.58–0.88)), while it does not significantly affect the risk of macrosomia (RCTs (RR = 0.29, 95% CI = 0.06–1.38), cohort studies (RR = 0.70, 95% CI = 0.30–1.62)), small for gestational age (SGA) infants (RCTs (RR = 1.18, 95% CI = 0.74–1.90)), or congenital hypothyroidism (CH) (cohort studies (RR = 1.27, 95% CI = 0.16–10.07)) in children. No significant difference in birth weight (RCTs (RR = 0.10, 95% CI = −0.04–0.24), cohort studies (RR = 0.10, 95% CI = −0.08–0.28)) was observed between the L-T4 treatment group and the non L-T4 treatment group. Regarding neonatal cord blood thyroid function, the TSH levels in the L-T4 treatment group were lower compared to the non L-T4 group (RCTs (RR = −2.48, 95% CI = −4.51–−0.45), cohort studies (RR = −3.53, 95% CI = −4.27 – −2.79)); however, no significant differences were found in FT3 (RCTs (RR = 0.06, 95% CI = −0.24–0.36), cohort studies (RR = 0.08, 95% CI = −0.72–0.88)) and FT4 levels (RCTs (RR = 0.07, 95% CI = −0.41–0.56), cohort studies (RR = 0.03, 95% CI = −1.18–1.24)) between the two groups.

**Conclusion:**

L-T4 treatment appears to reduce the incidence of preterm birth and LBWI in pregnant mothers with SCH, but it does not significantly affect the incidence of macrosomia, SGA, CH, or birth weight. Regarding the thyroid function in neonatal umbilical cord blood, L-T4 treatment in SCH pregnant women can reduce TSH levels in the umbilical cord blood of their newborns, while having no significant effect on FT3 and FT4 levels.

**Systematic Review Registration:**

https://www.crd.york.ac.uk/prospero/display_record.php?ID=CRD420251016450, PROSPERO CRD420251016450.

## Introduction

During pregnancy, the maternal body undergoes a distinct physiological state. In the first trimester, the placenta secretes human chorionic gonadotropin (HCG), which increases in concentration. Due to the structural similarity between HCG and thyroid-stimulating hormone (TSH), HCG can competitively bind to TSH receptors, thereby stimulating the synthesis and secretion of thyroid hormones, which leads to a temporary suppression of TSH levels. By the 10th week of gestation, HCG levels begin to decline, resulting in a gradual rise in TSH levels. Furthermore, the estrogen-induced synthesis of thyroxine-binding globulin (TBG), increased renal clearance of iodine, and heightened fetal iodine requirements contribute to a relatively insufficient thyroid reserve during pregnancy. These factors predispose pregnant women to subclinical hypothyroidism (SCH) (1–3). According to the 2022 Chinese Guidelines for the Prevention and Management of Thyroid Diseases in Pregnancy and Childbirth (4), SCH is characterized by a TSH concentration exceeding the upper limit of the pregnancy-specific reference range. In regions lacking a specific reference cut-off value, a TSH level greater than 4.0 mIU/L is utilized as the standard, while the free thyroxine (FT4) level remains within the normal range. SCH is a prevalent finding during pregnancy, with an incidence rate of approximately 2%–2.5% (5) in the United States and 7.33% (6) in China. Given that FT4 levels in patients with SCH typically remain within the normal range, the condition often presents in a compensatory state, rendering its onset subtle. As a result, the clinical manifestations are frequently atypical and may be overlooked. If SCH is not detected and managed early, it may progress to clinical hypothyroidism (hereinafter referred to as hypothyroidism), potentially resulting in various adverse pregnancy outcomes, including gestational diabetes, preeclampsia, miscarriage, preterm birth, low birth weight infants (LBWI), and neonatal respiratory distress syndrome, thereby impacting fetal growth and development (7, 8). Presently, existing guidelines and consensus statements have not definitively elucidated the clinical efficacy of levothyroxine (L-T4) in managing SCH during pregnancy. Consequently, this study employs a meta-analytic approach to examine the influence of L-T4 treatment for SCH in pregnant individuals on neonatal birth outcomes. The objective is to ascertain the therapeutic effectiveness of L-T4 in addressing SCH, thereby mitigating adverse perinatal outcomes and providing a theoretical foundation for early clinical intervention in SCH during pregnancy to enhance perinatal outcomes.

## Methods

### Literature inclusion and exclusion criteria

The inclusion criteria for this study include: the protocol for this review was recorded in the Prospective International Register of Systematic Reviews (PROSPERO) with the ID CRD 420251016450. The research adheres to the guidelines outlined in the Preferred Reporting Items for Systematic reviews and Meta-Analyses (PRISMA) statement (9). The search strategy was based on the PICOS (P: Population; I: Intervention; C: Comparison; O: Outcome; S: Study design) methodology. The PICOS framework is as follows: Population—pregnant women with singleton live births who were diagnosed with SCH during pregnancy; Intervention—received L-T4 treatment with no restrictions on the dosage or duration of the drug administration; Comparison—Placebo or a blank control; Outcome—occurrence of preterm labor (<37 weeks' gestation), LBWI (birth weight less than 2500 g), macrosomia (birth weight more than 4000 g) (10), congenital hypothyroidism (CH) (CH is defined as thyroid hormone deficiency present at birth.) (11), SGA (birth weight less than 10th percentile for gestational age), birth weight, and neonatal umbilical cord blood thyroid function, specifically TSH, free triiodothyronine (FT3), FT4 levels; and Study design—randomized clinical trials (RCTs) or case-control study.

The exclusion criteria for this study include: (1) Incomplete data where the full text is unavailable; (2) Conference abstracts, systematic evaluations, reviews, case reports,animal experiments, expert consensus, and master's and doctoral degree papers; (3) Duplicated publications; (4) Literature not in Chinese or English; and (5) low quality literature with questionable data authenticity.

### Literature search

A systematic review was conducted to examine the effects of L-T4 treatment of SCH in pregnancy on neonatal birth outcomes in databases including Literature searches were conducted PubMed, Embase, Web of Science, Cochrane Library, VIP Chinese Science and Technology Journal Database (VIP), China National Knowledge Internet (CNKI), Wan fang Database, and Sinomed, with search terms in both Chinese and English languages. The search strategy employed for this meta-analysis included keywords such as “Pregnancy” OR “Pregnancies” OR “Gestation” AND “subclinical” OR “sub-clinical” OR “subclin*” OR “sub-clin*” AND “hypothyroidisms” OR “Thyroid Stimulating Hormone Deficiency” OR “TSH Deficiency” AND “Thyroxine” OR “L-Thyroxine” OR “L-T4” AND “Therapeutics” OR “Treatments” OR “Therapy” up until June 2024. A combination search approach utilizing MeSH subject terms and free terms was employed, with references being systematically traced back to the included literature. The detailed search strategies for Cochrane, Pubmed are outlined in Supplementary Material.

### Data extraction

Two researchers (You ZY and Zhang YY) conducted independent screening of the literature for data extraction, cross-checking their findings. Any discrepancies were resolved through consensus discussion or consultation with a third party. The data extraction process encompassed: basic information of the included studies, including study title, first author, year of publication, study type, baseline characteristics, diagnostic criteria of study subjects, key elements of bias risk assessment, outcome indicators and outcome measures of interest.

### Quality evaluation

The quality of the included RCTs was assessed using the Cochrane Risk of Bias tool (http://handbook.cochrane.org), which checks for selection bias (random sequence generation), selection bias (allocation concealment), performance bias (blinding of participants and personnel), detection bias (blinding of outcome assessment), attrition bias (incomplete outcome data), reporting bias (selective reporting), other possible bias (bias from other sources). The quality of the literature was evaluated using the Newcastle-Ottawa Scale (NOS) (12) for cohort studies. The evaluation of cohort studies encompassed three primary dimensions: selection of study population, comparability between study groups, and measurement of outcomes, with scores on the NOS ranging from 0–9 points. Studies receiving a score of 6–9 were deemed to be of high quality, indicating a low risk of bias. Two researchers (You ZY and Zhang YY) independently conducted assessments, with a third researcher available to resolve any discrepancies.

### Statistical analysis

A meta-analysis was conducted using RevMan54 and Stata 14 software to systematically assess the effects of L-T4 treatment of SCH in pregnancy on neonatal birth outcomes. Mean ± standard deviation(MD) was utilized as the effect indicator for continuous data, while risk ratio (RR) was used for categorical data, with each effect size reported with a 95% confidence interval (CI). Heterogeneity was assessed through the application of the Chi-square test and *I*^2^ statistic. When *I*^2^ > 50% and *P* ≤ 0.l, it signified heterogeneity among the studies. In such cases, the random-effects model was employed to compute the combined statistic, and subgroup or sensitivity analyses were conducted to explore the underlying sources of heterogeneity. Conversely, if these criteria were not met, the fixed-effects model was utilized. Additionally, funnel plots were generated for the outcome measures, and publication bias was evaluated through the integration of Begg's test. There was significant difference between the two group when *P* ≤ 0.05.

## Results

### Results of the literature search and basic characteristics of the included studies

**Figure 1:**
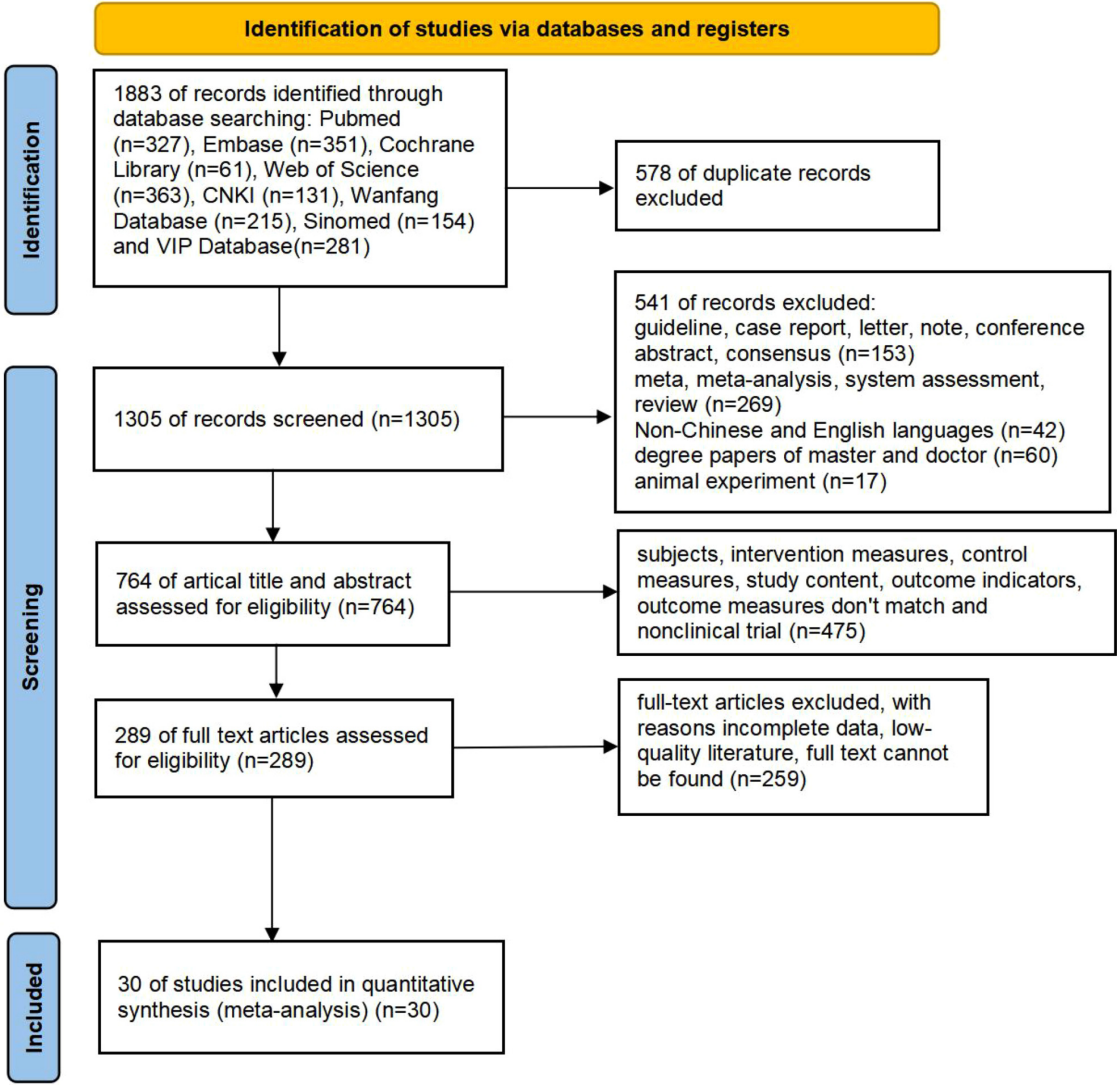
PRISMA flow diagram of study selection.

A total of 1883 articles were obtained, comprising 781 articles in Chinese (131 articles in CNKI, 215 articles in Wanfang database, 281 articles in VlP database, and 154 articles in Sinomed) and 1,102 articles in English (327 articles in PubMed, 351 articles in Embase, 61 articles in Cochrane library, 363 articles in Web of Science). 578 duplicates were removed, and 541 articles were excluded due to their meta, systematic evaluation, literature review, case report, expert consensus, animal test, etc. After reviewing titles, and abstracts, a total of 475 articles were excluded for non-compliance with intervention and control measures, study content, and outcome indicators. After reviewing full texts, an additional 259 articles were excluded for reasons such as inability to locate full text, incomplete data and low quality. Ultimately, 30 RCTs and cohort studies (13–42) were included in the literature screening process, as depicted in Figure 1. The 30 publications were published from 2013–2024 covering a total of 4 countries and regions, involving 5,578 cases in the group with L-T4 treatment (experimental group) and 9,024 cases in the group with non L-T4 treatment (control group). The basic information of the literature is shown in Supplementary Table S1.

### Literature quality assessment

In the analysis of 18 RCTs (13–30), 17 studies (13–28, 30) offered comprehensive descriptions of their randomization methods, while 11 studies (14–18, 22, 23, 25–28) sufficiently detailed their allocation concealment procedures. Only two studies (13, 14) reported employing double-blinding techniques. Blinding of outcome assessment was implemented in 14 studies (13–25, 28); however, one study demonstrated compromised data integrity (28). Furthermore, six studies (16, 18–20, 23, 24) did not explicitly address the issue of selective reporting of results, and one study (14) explicitly considered other potential sources of bias (Supplementary Figure S1). 12 cohort studies (31–42) were assessed for quality using the NOS, with three articles receiving a score of 6 (35, 38, 39) four articles receiving a score of 7 (32, 33, 41, 42), three articles receiving a score of 8 (31, 37, 40) and two articles receiving a score of 9 (34, 36), indicating high-quality literature. The quality scores of the included articles can be found in Supplementary Table S2.

### Meta-analysis

**Figure 2:**
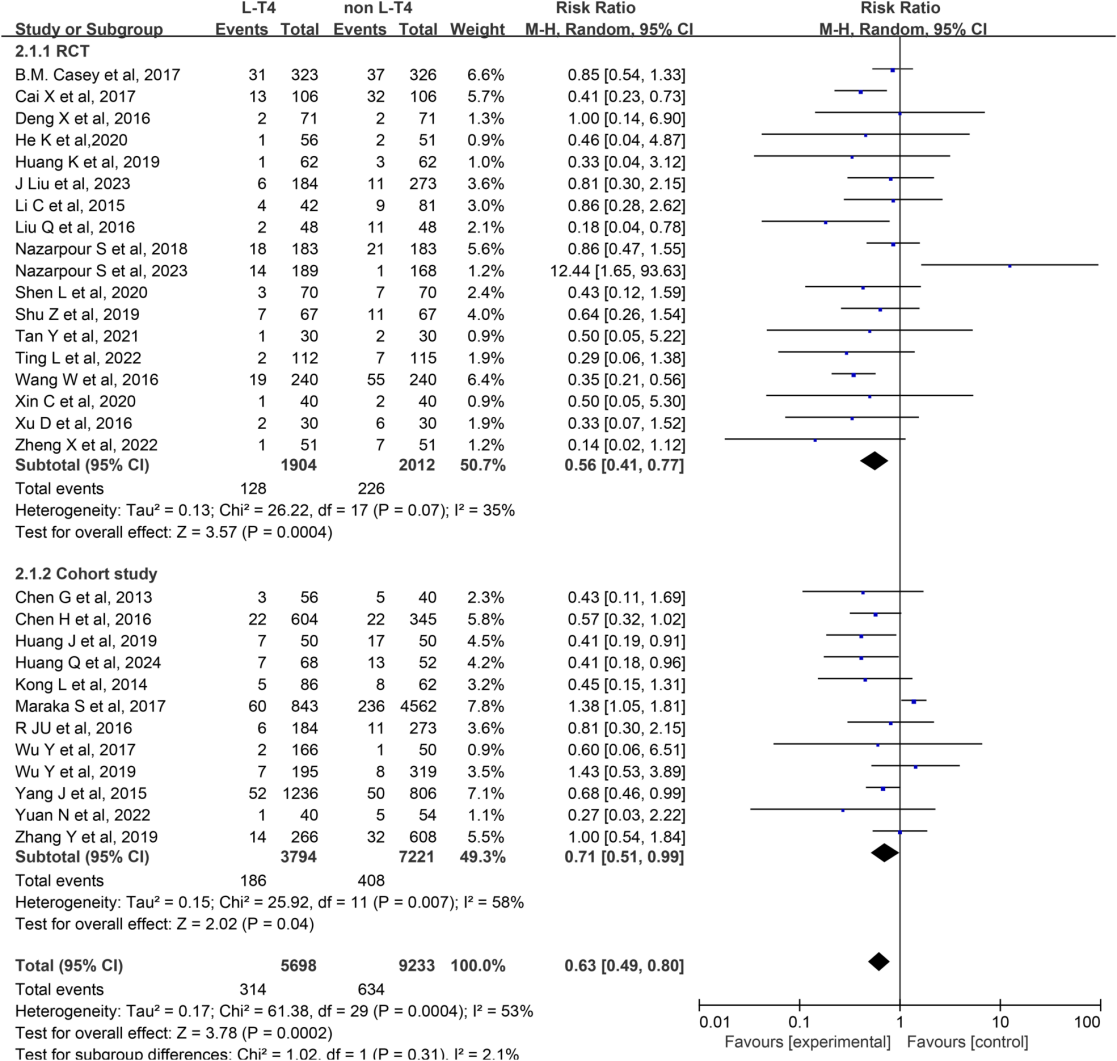
Forest plot for preterm.

Effect of L-T4 therapy for SCH in Pregnancy on preterm infants: An analysis of 30 studies (13–42) assessed the influence of L-T4 treatment for SCH during pregnancy on the occurrence of preterm birth. This body of research comprised 18 RCTs and 12 cohort studies. Due to statistical heterogeneity among the studies (*P* *=* 0.0004, *I*^2^ = 53%), a random-effects model was employed for the meta-analysis. The results indicated that L-T4 treatment for SCH in pregnant individuals may reduce the incidence of preterm birth in their offspring (RR = 0.63, 95% CI = 0.49–0.80. *P* *=* 0.0002). Subgroup analyses based on study design revealed that L-T4 therapy was linked to a reduced risk of preterm birth in the offspring of pregnant women with SCH, regardless of study design (RCTs (RR = 0.56, 95% CI = 0.41–0.77. *P* *=* 0.0004) or cohort study (RR = 0.71, 95% CI = 0.51–0.99. *P* = 0.04)) (Figure 2).

A subgroup analysis stratified by TPOAb status demonstrated that within the TPOAb-negative cohort, Six RCTs (RR = 0.61, 95% CI = 0.40–0.92. *P* = 0.02) and three cohort studies (RR = 0.51, 95% CI = 0.28–0.91. *P* = 0.02) showed L-T4 therapy was associated with a reduced incidence of preterm birth among offspring of pregnant women with SCH. In the TPOAb(±) group, 12 RCTs (RR = 0.89, 95% CI = 0.46–0.74. *P<*0.0001) similarly indicated a decreased likelihood of preterm birth with L-T4 therapy. In contrast, data from nine cohort studies (RR = 0.91, 95% CI = 0.76–1.09. *P* = 0.32) in this subgroup revealed no statistically significant difference in preterm birth risk between the L-T4 group and non-L-T4 group (Supplementary Tables S3, S4).

**Figure 3:**
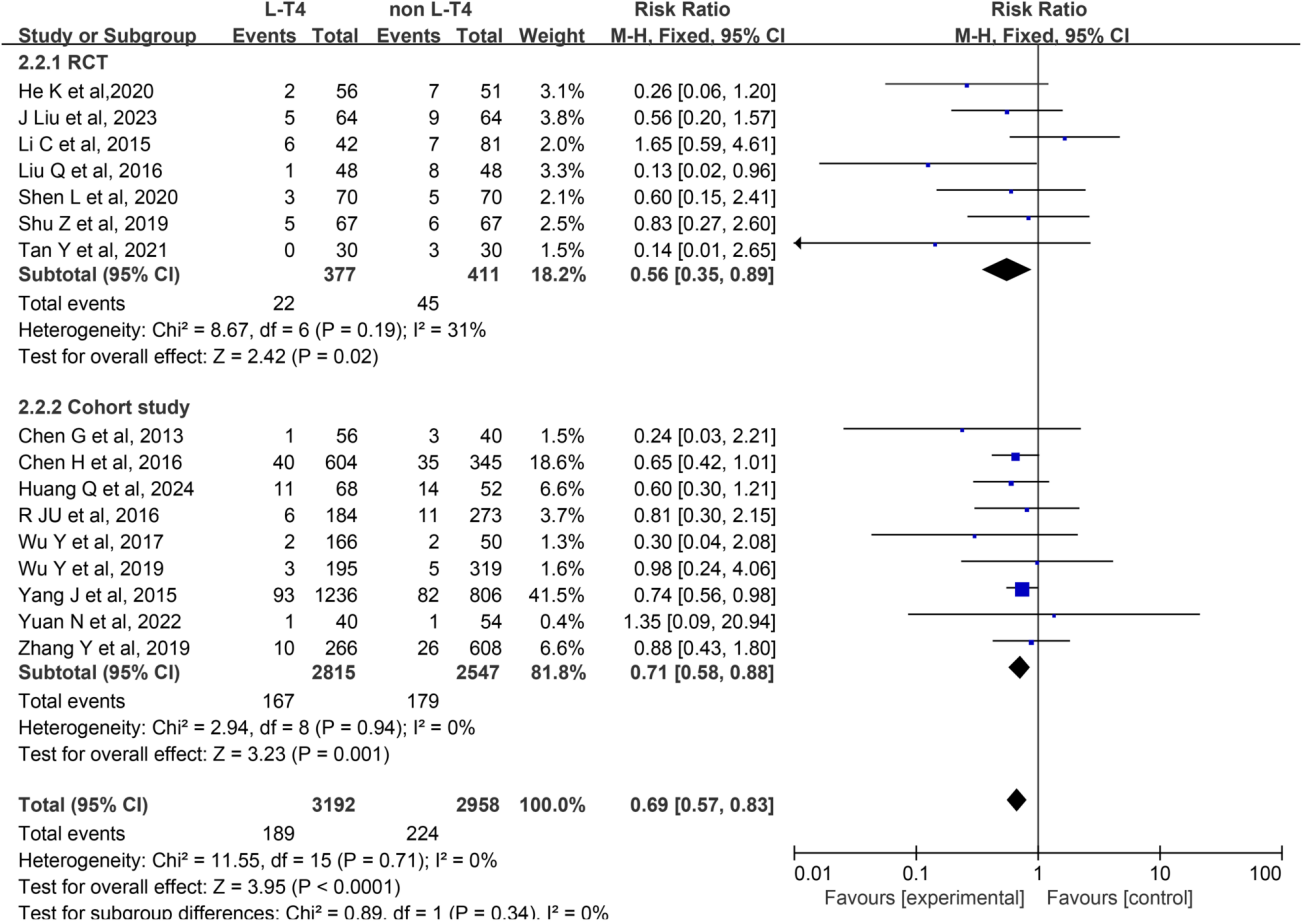
Forest plot for LBWI.

Impact of L-T4 Therapy for SCH in Pregnancy on LBWI: A analysis of 16 studies (13, 18, 19, 22, 23, 26, 29, 31–33, 36–38, 40–42), comprising seven RCTs and nine cohort studies, was conducted to evaluate the effect of L-T4 therapy for SCH during pregnancy on the incidence of LBWI. Due to the absence of statistical heterogeneity among these studies (*P* = 0.71, *I*^2^ = 0%), a fixed-effect model was employed for the meta-analysis. The findings indicated that neonates born to mothers who did not receive L-T4 treatment were at a significantly higher risk of developing LBWI compared to those whose mothers received L-T4 therapy (RR = 0.69, 95% CI = 0.57–0.83. *P* < 0.0001). Subgroup analysis based on study design revealed that L-T4 treatment effectively reduced the incidence of LBWI in both RCTs (RR = 0.56, 95% CI = 0.35–0.89. *P* = 0.02) and cohort study (RR = 0.71, 95% CI = 0.58–0.88. *P* *=* 0.001) groups (Figure 3).

A subgroup analysis stratified by TPOAb status showed that in the TPOAb(−) subgroup, neither three RCTs (RR = 0.54, 95% CI = 0.26–1.14. *P* = 0.1) nor two cohort studies (RR = 0.98, 95% CI = 0.66–1.44. *P* = 0.91) found a significant effect of L-T4 therapy on LBWI incidence in offspring of pregnant women with SCH. In the TPOAb(±) subgroup, four RCTs (RR = 0.57, 95% CI = 0.31–1.05. *P* = 0.07) also showed no statistically significant difference in LBWI incidence between the L-T4 and control groups. However, pooled data from seven cohort studies (RR = 0.71, 95% CI = 0.57–0.87. *P* = 0.001) suggested that L-T4 therapy might reduce the risk of LBWI (Supplementary Tables S3, S4).

**Figure 4:**
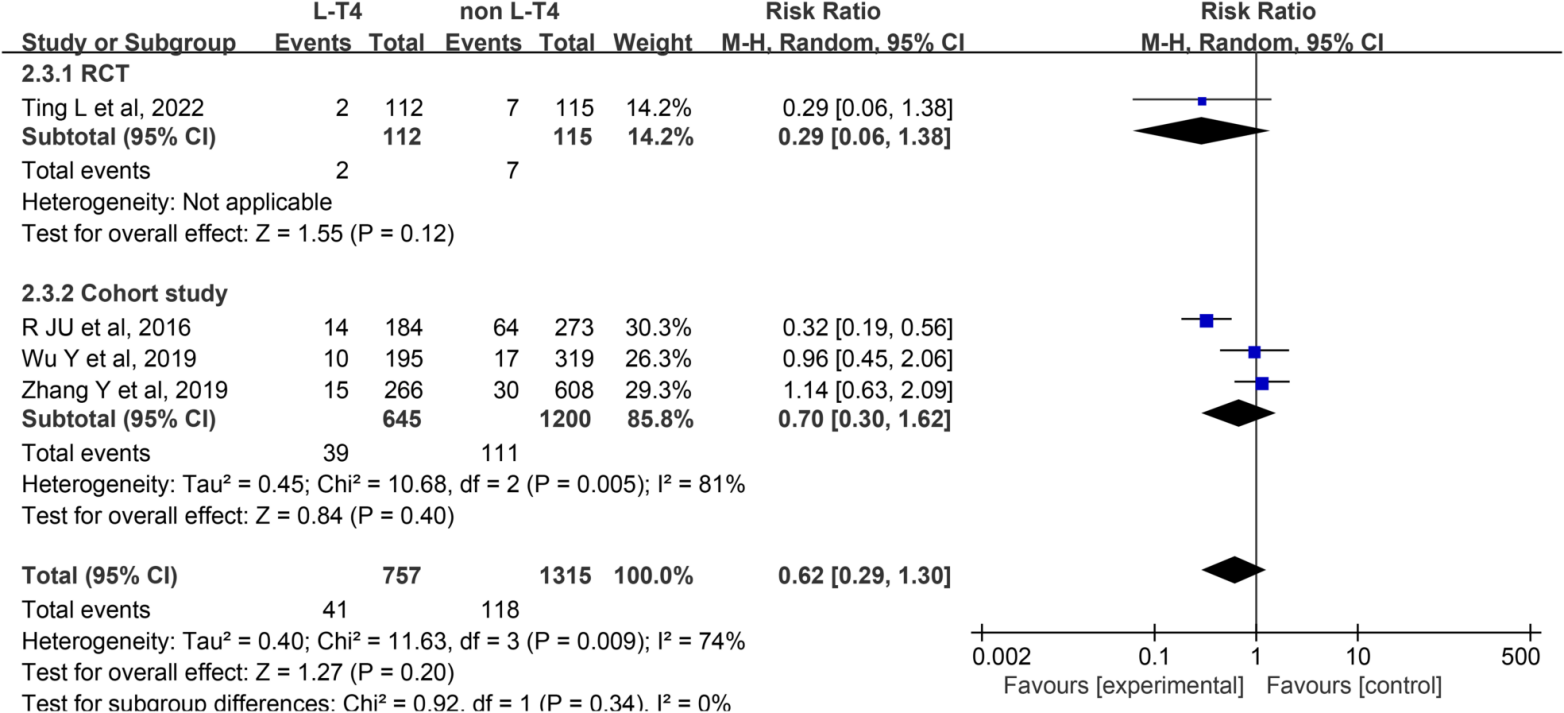
Forest plot for macrosomia.

Impact of L-T4 Therapy for SCH in Pregnancy on Macrosomia: An RCTs and three cohort studies (16, 31, 33, 37) have examined the impact of L-T4 treatment for SCH during pregnancy on the incidence of macrosomia. Due to statistical heterogeneity among these studies (*P* = 0.009, *I*^2^ = 74%), a random-effects model was employed for the meta-analysis. The findings indicated that L-T4 treatment did not significantly influence the risk of macrosomia in the offspring overall (RR = 0.62, 95% CI = 0.29–1.30. *P* = 0.20). Subgroup analyses based on study design revealed no statistically significant effect on the incidence of macrosomia in either the RCTs (RR = 0.29, 95% CI = 0.06–1.38. *P* = 0.12) or cohort study (RR = 0.70, 95% CI = 0.30–1.62. *P* = 0.40) groups (Figure 4).

A subgroup analysis stratified by TPOAb status revealed differing outcomes. Within the TPOAb negative cohort, one RCTs (RR = 0.29, 95% CI = 0.06–1.38. *P* = 0.12) demonstrated that L-T4 in pregnant women with SCH had no significant effect on the risk of macrosomia in their offspring. In contrast, a cohort study (RR = 0.32, 95% CI = 0.19–0.56. *P* < 0.001) suggested that L-T4 might reduce this risk. In the TPOAb(±) group, two cohort studies (RR = 1.07, 95% CI = 0.67–1.72. *P* = 0.78) indicated that L-T4 did not significantly affect the risk of macrosomia in offspring (Supplementary Tables S3, S4).

**Figure 5:**
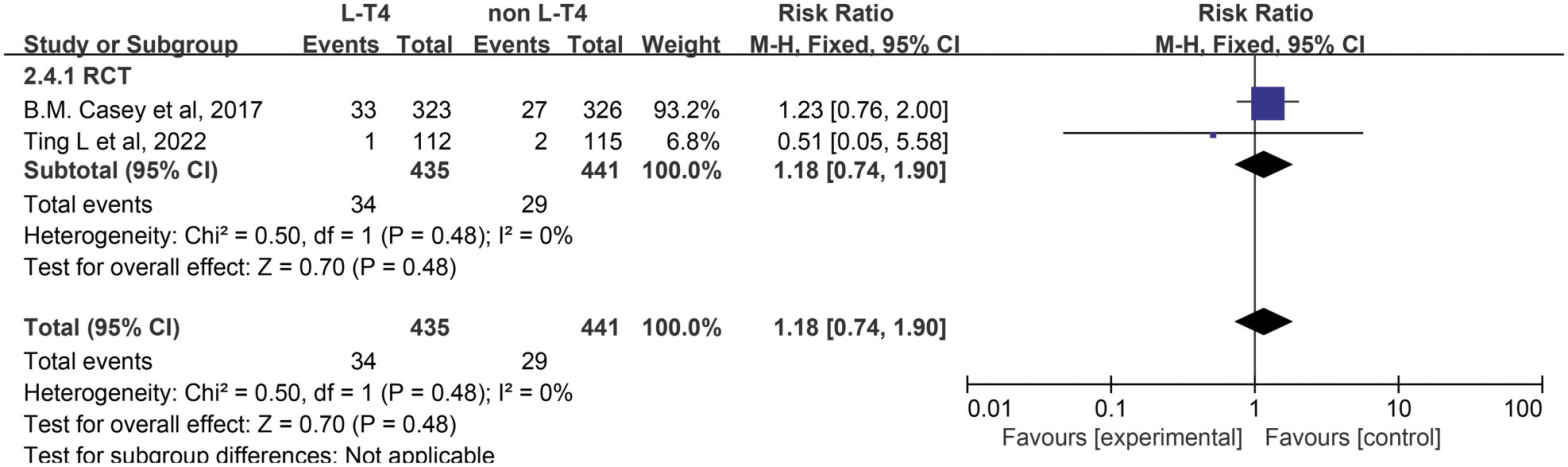
Forest plot for SGA.

The effect of L-T4 therapy for SCH during pregnancy on the incidence of SGA neonates has been investigated in two RCTs (14, 16). Given the lack of statistical heterogeneity between these studies (*P* = 0.48, *I*^2^ = 0%), a fixed-effect model was utilized for the meta-analysis. The results indicated no significant difference in the incidence of SGA neonates between the group receiving L-T4 treatment and the group not receiving L-T4 treatment among pregnant women with SCH (RR = 1.18, 95% CI = 0.74–1.90. *P* = 0.48) (Figure 5). Subgroup analyses stratified by TPOAb status indicated that there was no statistically significant impact on the incidence of SGA, irrespective of TPOAb status (Supplementary Tables S3, S4).

**Figure 6:**
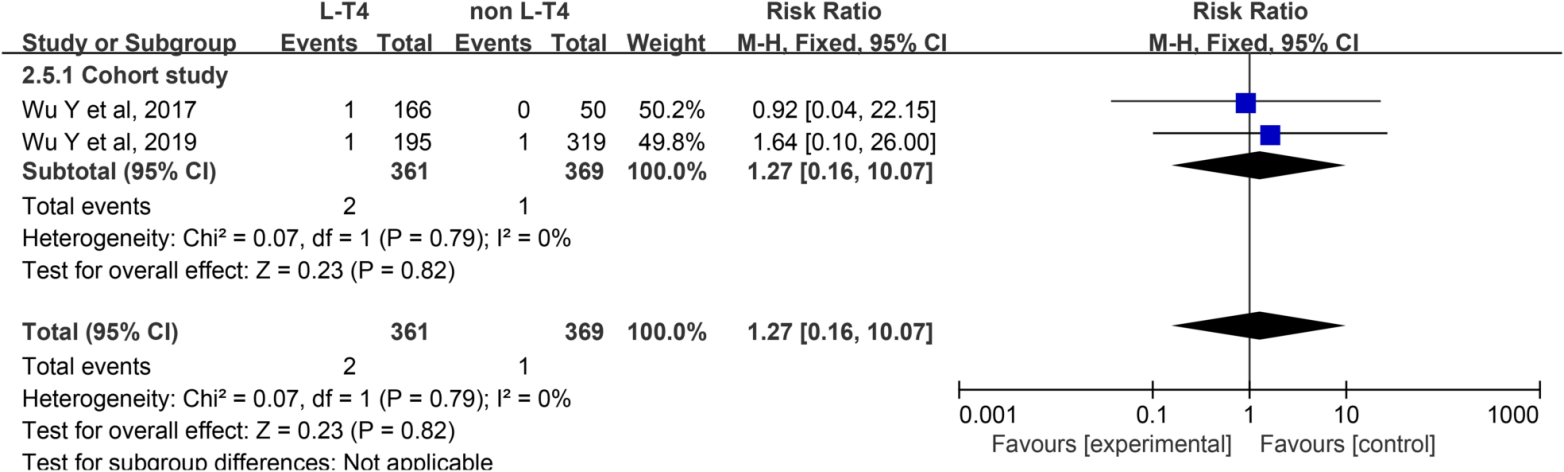
Forest plot for CH.

Impact of L-T4 Therapy for SCH in Pregnancy on CH: Two cohort studies (36, 37) have examined the impact of L-T4 treatment for SCH during pregnancy on the incidence of CH. Due to the absence of statistical heterogeneity among these studies (*P* = 0.79, *I*^2^ = 0%), a fixed-effect model was employed for the meta-analysis. There was no significant difference between the L-T4 group and the non-L-T4 group in the CH of neonates born to SCH pregnant womens (RR = 1.27, 95% CI = 0.16–10.07. *P* = 0.82) (Figure 6). Subgroup analyses based on TPOAb status revealed no statistically significant effect on the incidence of CH, regardless of TPOAb status (Supplementary Tables S3 and S4).

**Figure 7:**
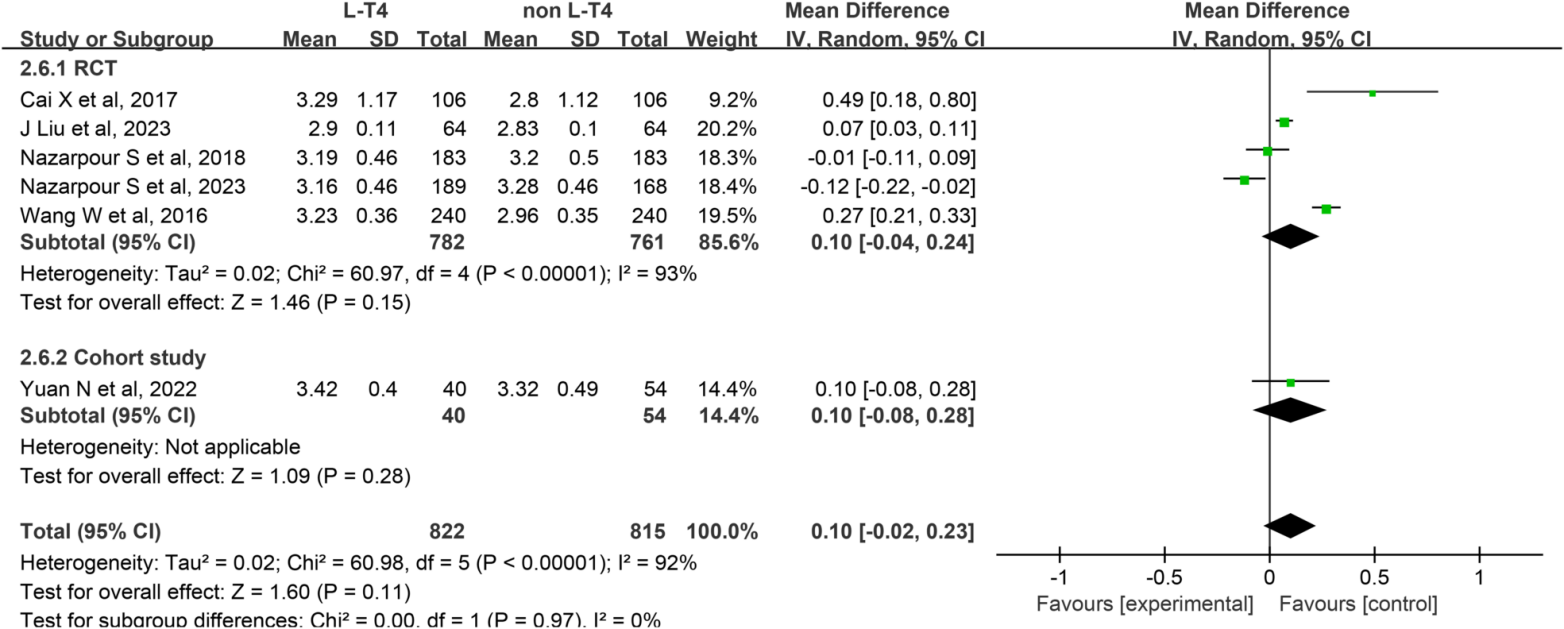
Forest plot for birth weight.

Impact of L-T4 Therapy for SCH in Pregnancy on birth weight: Six studies (13, 15, 17, 21, 25, 32) have examined the impact of L-T4 treatment for SCH during pregnancy on the incidence of birth weight. These studies comprise five RCTs and one cohort study. Due to the presence of statistical heterogeneity among these studies (*P* < 0.0001, *I*^2^ = 92%), a random-effects model was employed for the meta-analysis.The analysis revealed no significant difference in the birth weight of neonates born to SCH pregnant women between the L-T4 treatment group and the non-L-T4 group (RR = 0.10, 95% CI = −0.02–0.23. *P* = 0.11). Subgroup analyses stratified by study design revealed no statistically significant differences in neonatal birth weight between the L-T4 treatment group and the control group, regardless of whether the studies were RCTs (RR = 0.10, 95% CI = −0.04–0.24. *P* = 0.15) or cohort studies (RR = 0.10, 95% CI = −0.08–0.28. *P* *=* 0.28) (Figure 7).

A subgroup analysis stratified by TPOAb status demonstrated that, within the TPOAb-negative group, both an RCTs (RR = −0.01, 95% CI = −0.11–0.09. *P* *=* 0.84) and a cohort study (RR = 0.10, 95% CI = −0.08–0.28. *P* *=* 0.28) found no significant difference in birth weight of offspring between pregnant women with SCH receiving L-T4 and those in the control group. Similarly, in the TPOAb(±) group, findings from four RCTs corroborated the absence of a statistically significant difference in birth weight between the L-T4-treated and control groups (RR = 0.14, 95% CI = −0.03–0.31. *P* *=* 0.11) (Supplementary Tables S3, S4).

**Figure 8:**
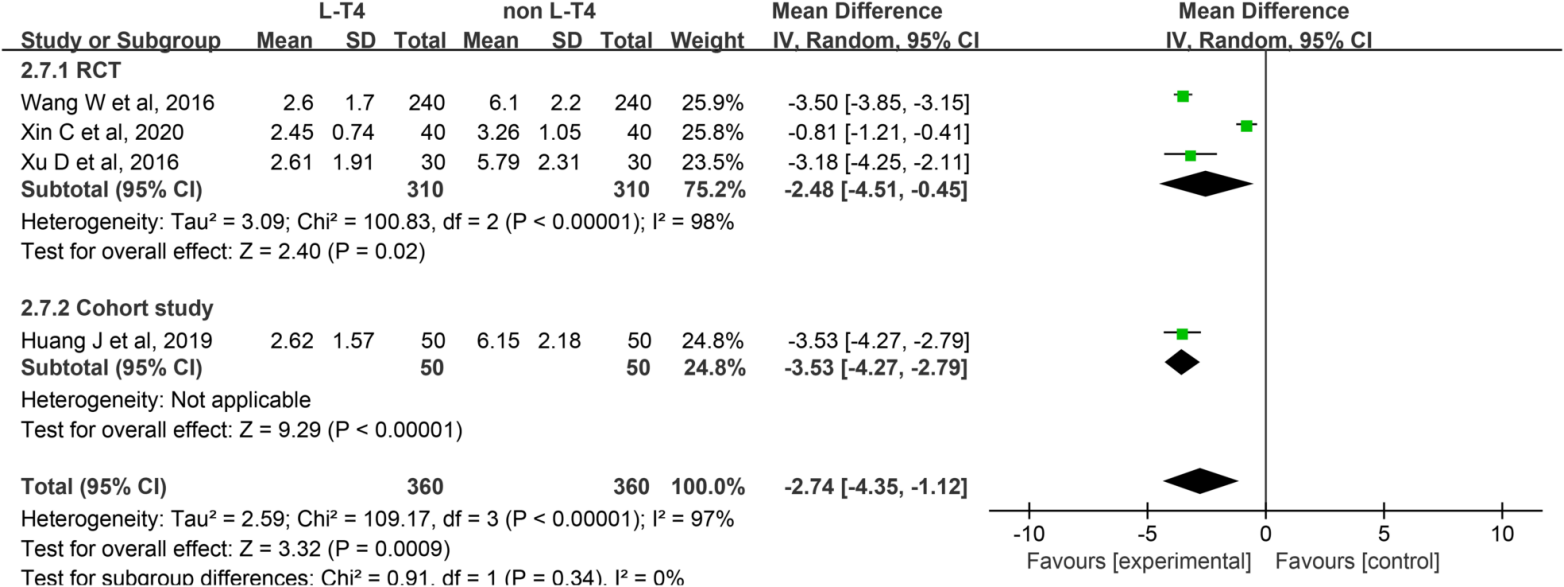
Forest plot for neonatal cord blood TSH.

Impact of L-T4 Therapy for SCH in Pregnant Women on Neonatal Cord Blood TSH Levels: A total of four studies (20, 25, 30, 39) have investigated the impact of L-T4 therapy on SCH during pregnancy concerning neonatal cord blood TSH levels. These studies comprise three RCTs and one cohort study. Due to statistical heterogeneity among these studies (*P* < 0.0001, *I*^2^ = 97%), a random-effects model was employed for the meta-analysis. The findings indicated that L-T4 treatment in pregnant women with SCH is associated with a reduction in TSH levels in the umbilical cord blood of their neonates (RR = −2.74, 95% CI = −4.35–−1.12. *P* *=* 0.0009). Furthermore, subgroup analysis revealed that L-T4 therapy significantly decreases TSH levels in both the RCTs (RR = −2.48, 95% CI = −4.51–−0.45. *P* *=* 0.02) and the cohort study (RR = −3.53, 95% CI = −4.27–−2.79. *P* < 0.0001) (Figure 8).

A subgroup analysis stratified by TPOAb status revealed that, within the TPOAb-negative cohort, both an RCTs (RR = −3.18, 95% CI = −4.25–−2.11. *P* < 0.001) and a cohort study (RR = −3.53, 95% CI = −4.27–−2.79. *P* < 0.001) demonstrated that L-T4 treatment was associated with a reduction in cord blood TSH levels in the offspring of pregnant women with SCH. In contrast, in the TPOAb(±) group, two RCTs found no statistically significant differences in cord blood TSH levels between the L-T4-treated and control groups (RR = −2.16, 95% CI = −4.79–0.48. *P* *=* 0.11) (Supplementary Tables S3, S4).

**Figure 9:**
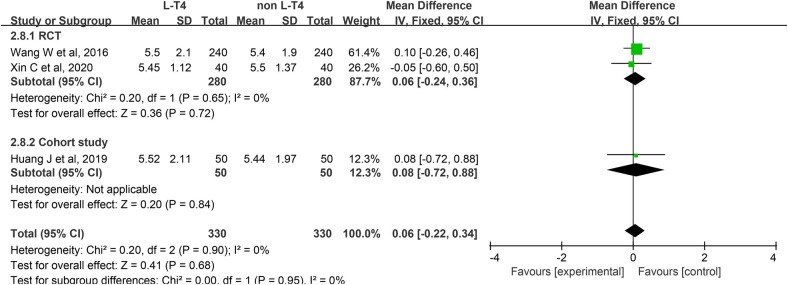
Forest plot for neonatal cord blood FT3.

Impact of L-T4 Therapy for SCH in Pregnant Women on Neonatal Cord Blood FT3 Levels: Three studies (20, 25, 39), comprising two RCTs and one cohort study, have investigated the impact of L-T4 therapy on SCH during pregnancy concerning neonatal cord blood FT3 levels. Due to the absence of statistical heterogeneity among these studies (*P* = 0.90, *I*^2^ = 0%), a fixed-effect model was employed for the meta-analysis. The findings indicated that L-T4 treatment did not significantly influence the risk of FT3 levels in the umbilical cord blood of their neonates (RR = 0.06, 95% CI = −0.22–0.34. *P* = 0.68). Subgroup analyses further indicated no significant differences in cord blood FT3 levels between the L-T4 and non-L-T4 groups in both the RCTs (RR = 0.06, 95% CI = −0.24–0.36. *P* = 0.72) and the cohort study (RR = 0.08, 95% CI = −0.72–0.88. *P* = 0.84) (Figure 9). Additionally, subgroup analyses showed no significant differences in the FT3 levels in cord blood between the L-T4 and non-L-T4 groups among pregnant women with TPOAb(±) (RR = 0.06, 95% CI = −0.24–0.36. *P* = 0.72) or TPOAb(−) (RR = 0.08, 95% CI = −0.72–0.88. *P* = 0.84) SCH (Supplementary Tables S3, S4).

**Figure 10:**
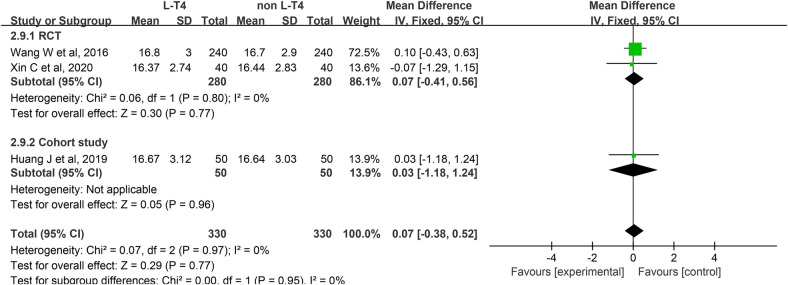
Forest plot for neonatal cord blood FT4.

Impact of L-T4 Therapy for SCH in Pregnant Women on Neonatal Cord Blood FT4 Levels: Three studies (20, 25, 39), comprising two RCTs and one cohort study, have investigated the impact of L-T4 therapy on SCH during pregnancy concerning neonatal cord blood FT4 levels. Due to the absence of statistical heterogeneity among these studies (*P* = 0.97, *I*^2^ = 0%), a fixed-effect model was employed for the meta-analysis. The findings indicated that L-T4 treatment did not significantly influence the risk of FT4 levels in the umbilical cord blood of their neonates (RR = 0.07, 95% CI = −0.38–0.52. *P* = 0.77). Subgroup analysis revealed that there was no significant difference in the level of FT4 in cord blood between the L-T4 group and the non-L-T4 group within both the RCTs (RR = 0.07, 95% CI = −0.41–0.56. *P* = 0.77) and the cohort study (RR = 0.03, 95% CI = −1.18–1.24. *P* = 0.96) (Figure 10). Furthermore, subgroup analysis revealed that there was no statistically significant difference in the FT4 level in cord blood between the L-T4 group and non-L-T4 group among pregnant mothers with TPOAb(±) (RR = 0.07, 95% CI = −0.41–0.56. *P* = 0.77) or TPOAb(−) (RR = 0.03, 95% CI = −1.18–1.24. *P* = 0.96) SCH (Supplementary Tables S3, S4).

### Publication bias and sensitivity analysis

We employed inverted funnel plot analyses for each outcome to assess publication bias among the studies (Supplementary Figure S2A-I). The results indicated that the distribution of points in the funnel plots was largely symmetrical, suggesting a minimal likelihood of publication bias. Furthermore, Begger's test revealed no significant evidence of publication bias (all *P* > 0.05) (supplementary Figure S3A-I). Excluding any single study from the meta-analyses, with the exception of the SGA outcome, had an insignificant impact on the overall results. This finding implies minimal inter-study variability and a low reliance on any individual study, thereby affirming the robustness of the analytical outcomes. The potential lack of robustness in the SGA meta-analysis may be due to the limited number of studies included (supplementary Figure S4A-I).

## Discussions

During pregnancy, the heightened demand for thyroid hormones, elevated levels of TBG, and fluctuations in TSH induced by HCG collectively contribute to a relative insufficiency in thyroid reserve among pregnant women. This physiological change may predispose individuals to SCH during pregnancy, which, if inadequately managed, can lead to various complications (3, 43, 44). According to the 2014 guidelines issued by the European Thyroid Society regarding SCH in pregnant women and children, SCH during pregnancy, particularly in the first trimester, is associated with an elevated risk of adverse outcomes such as preterm birth, LBWI, and SGA infants (45). In 2019, an assessment conducted at Boston Medical Center involving 8,413 pregnant women revealed that maternal serum TSH levels exceeding 4 mIU/L were correlated with an elevated risk of preterm birth (RR = 2.17, 95% CI 1.15–4.07, *P* = 0.016) and respiratory distress syndrome (RR = 2.83, 95% CI 1.02–7.86, *P* = 0.046) in their offspring. However, these levels did not demonstrate statistical significance concerning preeclampsia/eclampsia and adverse pregnancy outcomes associated with LBWI (46). In contrast, Sitori et al. (47) identified a significant association between SCH in pregnant women and an increased risk of preeclampsia and LBWI in their investigations into the effects of SCH and autoimmune hypothyroidism on pregnancy outcomes. Recent research examining the association between TSH levels and pregnancy outcomes in patients with SCH has demonstrated that the incidence of preterm birth, miscarriage, and SGA infants is significantly higher in patients with poorly controlled SCH compared to those with well-managed SCH (*P* < 0.05) (48). This article incorporated thirty RCTs and cohort studies, all of which demonstrated high-quality scores. The results of meta-analysis revealed that treatment with L-T4 in SCH mothers significantly reduced the incidence of preterm birth and LBWI. However, no significant differences were observed in the incidence of SGA, macrosomia, and birth weight between the L-T4 treatment group and the non-L-T4 treatment group. Consequently, the administration of L-T4 for the treatment of SCH during pregnancy appears to effectively decrease the incidence of preterm births and LBWI among offspring. However, the impact of L-T4 on the incidence of SGA, macrosomia, and birth weight in neonates born to mothers with SCH requires further investigation.

Recent research indicates that autoimmune hypothyroidism is the predominant cause of hypothyroidism during pregnancy, particularly in regions with adequate iodine levels, where Hashimoto's thyroiditis is most prevalent. Laboratory assessments typically reveal the presence of TPOAb or TBAb, which have the potential to influence fetal and neonatal thyroid function via the placental barrier (49). However, the impact of maternal hypothyroidism on neonatal thyroid function post-birth remains inadequately understood. The investigation highlighted that pregnant women with TPOAb and TBAb can impair neonatal thyroid function via the placental barrier starting from the second trimester, leading to transient clinical hypothyroidism (TCH) in neonates (50, 51). A study conducted by Rodriguez et al. in 2017 demonstrated a strong correlation between maternal TPOAb levels and those in their newborns (Spearman: 0.89, *P* < 0.001). However, the study was unable to establish a correlation between maternal TSH and TPOAb levels and the TSH levels in their offspring (49). In their investigation of mothers with autoimmune thyroid disease, Dussault and Fisher (52) identified a significantly higher prevalence of neonatal TCH (27% vs. 15%, *P* = 0.04). In neonates with hypothyroidism, they observed that elevated TSH concentrations were more frequently associated with maternal thyroid dysfunction (7.0% vs. 0.9%, *P* < 0.001). In 2022, at the Ha'Emek Pediatric Medical Center, 496 neonatal heel blood screenings were conducted on infants born to mothers with abnormal thyroid function (among them, 91.4% of pregnant women were diagnosed with hypothyroidism). Among these, 87 cases exhibited TSH levels exceeding 10 mIU/L, with 3 cases subsequently diagnosed with CH, representing an incidence rate of 6 per 1,000. This rate is notably higher than the incidence of CH in the general population of European and American countries, which ranges from 1 in 4,000 to 1 in 2,000 (53). The study demonstrated that treatment of pregnant mothers with SCH using L-T4 could effectively reduce TSH levels in the umbilical cord blood of their offspring. The subgroup analysis indicated that L-T4 treatment effectively reduced the TSH levels in the umbilical cord blood of neonates born to pregnant mothers with SCH who were TPOAb(−). Conversely, no significant difference was observed in the TSH levels in cord blood between the L-T4 treated group and the non L-T4 treated group among pregnant mothers with TPOAb(±) SCH, corroborating the preceding findings. However, there is a paucity of research examining the effects of L-T4 on CH, FT4, and FT3 in the management of SCH during pregnancy. Additionally, enhancing the detection of thyroid function antibodies in pregnant women and their newborns remains challenging in clinical practice. Consequently, there is a need for multi-center and large-sample studies to elucidate the impact of various thyroid function parameters during pregnancy on neonatal thyroid function postpartum, with particular emphasis on neonatal CH, FT3, and FT4 levels. Such research is essential to improve pregnancy outcomes in cases of maternal comorbidities.

## Limitations

This study has several limitations. Firstly, the literature reviewed was restricted to publications in Chinese and English. Although efforts were made to limit the sample size of the included studies, some studies still featured small sample sizes. Consequently, our meta-analysis incorporated both RCTs and cohort studies. It must be explained that, To address this limitation, we have meticulously categorized and delineated the results of both RCTs and non-RCTs within this article, with the objective of augmenting the clarity and reference value of our findings. Secondly, variations in study parameters, such as the definition of SCH in pregnancy, participant age, duration of intervention, presence of thyroid TBAb, and ethnicity, may introduce selection bias. Furthermore, this study did not investigate the effects of L-T4 treatment on neurological development due to the limited research available on the association between maternal SCH and intellectual disabilities in children, such as low IQ, language delay, and global developmental delay. The diversity of assessment scales employed to evaluate postnatal children's intellectual development and cognitive function further complicates this issue, as the heterogeneity in assessment methods across studies leads to a lack of uniformity and comparability in the findings. Consequently, it is imperative to explore this outcome through investigations with higher methodological quality and more standardized evaluation techniques. Finally, this study excluded abstracts and conference papers, potentially contributing to publication bias. Despite utilizing funnel plots and Begger's test to mitigate this bias, future research should systematically seek out grey literature or directly engage with authors to acquire unpublished data or detailed results. Such efforts are essential to further minimize publication bias and enhance the reliability of the conclusions drawn.

## Conclusions

In summary, L-T4 therapy has been shown to decrease the incidence of preterm birth and LBWI in pregnant women diagnosed SCH. However, it does not significantly affect the incidence of macrosomia, SGA, CH, or birth weight. Regarding the thyroid function in neonatal umbilical cord blood, L-T4 treatment in SCH pregnant women results in a reduction of TSH levels in the umbilical cord blood of their newborns, while having no significant impact on FT3 and FT4 levels.Given the limited sample size of the study, it remains essential to expand the sample and conduct a multicenter investigation to ascertain the impact of L-T4 on birth outcomes in pregnancies. Consequently, regular monitoring of thyroid function in pregnant women is warranted, along with the implementation of appropriate interventions to enhance pregnancy outcomes.

## Data Availability

The original contributions presented in the study are included in the article/Supplementary Material, further inquiries can be directed to the corresponding authors.
