# Supplementary material for: Meta-analysis of the effects of levothyroxine therapy for subclinical hypothyroidism during pregnancy on offspring outcomes
Source: Front Pediatr. 2025 Jul 10;13:1530859. doi: 10.3389/fped.2025.1530859 (PMC12287073; doi:10.3389/fped.2025.1530859)
Supplement: Supplementary file 1 [file Datasheet1.docx]

Supplementary Material

# Supplementary Figures

**Figure 1** Risk of bias analysis of RCTs according to RoB 2

#

**Figure 2** funnel plot analysis

| **** | **** | **** |
| --- | --- | --- |
| Figure 2A Funnel plot for preterm | Figure 2B Funnel plot for LBWI | Figure 2C Funnel plot for macrosomia |

| **** | **** | **** |
| --- | --- | --- |
| Figure 2D Funnel plot for SGA | Figure 2E Funnel plot for CH | Figure 2F Funnel plot for birth weight |
| **** | **** | **** |
| Figure 2G Funnel plot for cord blood TSH | Figure 2H Funnel plot for cord blood FT3 | Figure 2I Funnel plot for cord blood FT4 |

**Figure 3** Begger’s tests

| **** | **** | **** |
| --- | --- | --- |
| Figure 3A Begger’s test for preterm | Figure 3B Begger’s test for LBWI | Figure 3C Begger’s test for macrosomia |

| **** | **** | **** |
| --- | --- | --- |
| Figure 3D Begger’s test for SGA | Figure 3E Begger’s test for CH | Figure 3F Begger’s test for birth weight |
| **** | **** | **** |
| Figure 3G Begger’s test for cord blood TSH | Figure 3H Begger’s test for cord blood FT3 | Figure 3I Begger’s test for cord blood FT4 |

**Figure 4** Sensitivity analysis

| **** | **** |
| --- | --- |
| Figure 4A Sensitivity analysis for preterm | Figure 4B Sensitivity analysis for LBWI |
| **** | **** |
| Figure 4C Sensitivity analysis for macrosomia | Figure 4D Sensitivity analysis for SGA |

|  |  |
| --- | --- |
| Figure 4E Sensitivity analysis for CH | Figure 4F Sensitivity analysis for birth weight |

|  |  |
| --- | --- |
| Figure 4G Sensitivity analysis for cord blood TSH | Figure 4H Sensitivity analysis for cord blood FT3 |

|  |  |
| --- | --- |
| Figure 4I Sensitivity analysis for cord blood FT4 |  |

# Supplementary Tables

**Table 1** Basic characteristics of the included literature

| References | Country | Design | Definition of SCH | TPOAb status | Initial intervention  period | L-T4 initial therapeutic dose | Sample size | |
| --- | --- | --- | --- | --- | --- | --- | --- | --- |
|  |  |  |  |  |  |  | Experimental | Control |
| R JU et,2016 | Beijing,  China | Prospective  cohort  study | 97.5th percentile＜TSH levels；  2.5th percentile＜FT4 levels＜97.5th percentile | TPOAb(-) | T1 | - | 184 | 273 |
| Maraka S et,2017 | America | Retrospective  cohort  study | 2.5mIU/L＜TSH＜10mIU/L | TPOAb(±) | T1 | 50ug  (25ug-62.5ug) | 843 | 4562 |
| Zhang Y et,2019 | Beijing,  China | Retrospective  cohort  study | 2.5mIU/L＜TSH＜10mIU/L | TPOAb(±) | - | - | 266 | 608 |
| Yuan N et,2022 | Beijing,  China | Prospective  cohort  study | 4mIU/L＜TSH | TPOAb(-) | T1 | - | 40 | 54 |
| J Liu et,2023 | Gansu,  China | RCT | 2.5mIU/L＜TSH＜10mIU/L | TPOAb(±) | - | 50ug/75ug | 64 | 64 |
| B.M. Casey et,2017 | Washington, USA | RCT | 4mIU/L＜TSH，0.86ng/dl＜FT4＜1.9ng/dl | TPOAb(±) | T1 | 100ug | 323 | 326 |
| Nazarpour S et,2023 | Iran | RCT | 2.5mIU/L＜TSH＜10mIU/L | TPOAb(±) | T1 | 1ug/kg/d | 189 | 168 |
| Nazarpour S et,2018 | Iran | RCT | 2.5mIU/L＜TSH＜10mIU/L  1ng/dl＜FT4＜4.5ng/dl | TPOAb(-) | T2、T3 | 1ug/kg/d | 183 | 183 |
| Ting L et,2022 | Jiangsu,  China | RCT | 2.5mIU/L＜TSH＜10mIU/L  0.59ng/dl＜FT4＜1.25ng/dl | TPOAb(-) | T1 | 50ug | 112 | 115 |
| Li C et,2015 | Zhejiang,  China | RCT | 2.57mIU/L＜TSH，8.23pmol/L≤FT4≤13.79pmol/L | TPOAb(±) | T1 | 50ug | 42 | 81 |
| Liu Q et,2016 | Hebei,  China | RCT | 2.5mIU/L＜TSH＜10mIU/L | TPOAb(±) | T1 | 25ug | 48 | 48 |
| Kong L et,2014 | Jiangsu,  China | Retrospective  cohort  study | 2.5mIU/L＜TSH＜10mIU/L | TPOAb(±) | T1 | 12.5ug | 86 | 62 |
| Wu Y et,2017 | Shanghai,  China | Retrospective  cohort  study | T1: 2.5mIU/L＜TSH＜10mIU/L  T2 T3: 3mIU/L＜TSH＜10mIU/L | TPOAb(±) | T1-T3 | - | 166 | 50 |
| Wu Y et,2019 | Shanghai,  China | Retrospective  cohort  study | T1: 2.5mIU/L＜TSH＜10mIU/L  T2 T3: 3mIU/L＜TSH＜10mIU/L | TPOAb(±) | T1-T3 | - | 195 | 319 |
| Chen G et,2013 | Shanxi,  China | Prospective  cohort  study | T1: 2.5mIU/L＜TSH＜10mIU/L  T2 T3: 3mIU/L＜TSH＜10mIU/L | TPOAb(±) | T1 | 25-100ug | 56 | 40 |
| Xin C et,2020 | Hebei,  China | RCT | T1：2.5mIU/L＜TSH  T2 T3：3mIU/L＜TSH | TPOAb(±) | T1-T3 | 50ug-100ug | 40 | 40 |
| Cai X et,2017 | Hubei,  China | RCT | 2.5mIU/L＜TSH＜10mIU/L | TPOAb(±) | T1-T3 | 50ug | 106 | 106 |
| Tan Y et,2021 | Guangxi,  China | RCT | 2.5mIU/L＜TSH＜10mIU/L | TPOAb(±) | T1 | 50ug | 30 | 30 |
| Huang J et,2019 | Sichuan,  China | Retrospective  cohort  study | TSH＞4.20mU/L，12.00pmol/L＜FT4＜22.00pmol/L | TPOAb(-) | T1 | 25-50ug | 50 | 50 |
| Shen L et,2020 | Jiangsu,  China | RCT | 2.5mIU/L＜TSH，FT4＜11.5pmol/L | TPOAb(-) | - | 25-50ug | 70 | 70 |
| Zheng X et,2022 | Zhejiang,  China | RCT | - | TPOAb(±) | - | 50-100ug | 51 | 51 |
| Wang W et,2016 | Zhejiang,  China | RCT | T1：2.5mIU/L＜TSH；  T2 T3：3mIU/L＜TSH | TPOAb(±) | T1-T3 | 50-75ug | 240 | 240 |
| Shu Z et,2019 | Hebei,  China | RCT | 97.5th percentile＜TSH;  2.5th percentile＜FT4＜97.5th percentile | TPOAb(-) | T1-T3 | 50ug | 67 | 67 |
| Deng X  et,2016 | Guangdong,  China | RCT | 2.5mIU/L＜TSH＜10mIU/L | TPOAb(±) | T1、T2 | 25-100ug | 71 | 71 |
| Huang K et,2019 | Jiangsu,  China | RCT | 2.5mIU/L＜TSH＜10mIU/L | TPOAb(±) | - | 13-50ug | 62 | 62 |
| Chen H et,2016 | Xiamen,  China | Retrospective  cohort  study | 2.5mIU/L＜TSH | TPOAb(±) | T1 | 25ug | 604 | 345 |
| He K  et,2020 | Hebei,  China | RCT | 4mIU/L＜TSH | TPOAb(-) | T1 | 25ug-50ug | 56 | 51 |
| Yang J  et,2015 | Henan,  China | Retrospective  cohort  study | 5.22mIU/L＜TSH＜10.00mIU/L,12.91pmol/l＜FT4＜22.35pmol/l | TPOAb(±) | T1 | - | 1236 | 806 |
| Huang Q et,2024 | Hubei,  China | Retrospective  cohort  study | 2.5mIU/L＜TSH＜10mIU/L | TPOAb(±) | - | 25-100ug | 68 | 52 |
| Xu D  et,2016 | Jilin,  China | RCT | 4.94mIU/L＜TSH  12.0pmol/L＜FT4＜22.0pmol/L | TPOAb（-） | - | 50-100ug | 30 | 30 |

**Table 2** NOS score of cohort studies included in this meta-analysis

| **References** | **Selection** | | | | **Comparability** | | **Exposure** | | | **Total** |
| --- | --- | --- | --- | --- | --- | --- | --- | --- | --- | --- |
|  | **①** | **②** | **③** | **④** |  |  | **①** | **②** | **③** |  |
| R JU et, 2016 | ★ | ★ | ★ | ★ | ★ | |  | ★ | ★ | 7 |
| Maraka S et, 2017 | ★ | ★ | ★ | ★ | ★★ | | ★ | ★ | ★ | 9 |
| Zhang Y et, 2019 | ★ | ★ | ★ | ★ | ★★ | | ★ |  | ★ | 8 |
| Yuan N et, 2022 | ★ | ★ | ★ | ★ | ★★ | |  |  | ★ | 7 |
| Kong L et, 2014 | ★ | ★ | ★ | ★ | ★ | |  |  | ★ | 6 |
| Wu Y et, 2017 | ★ | ★ | ★ | ★ | ★★ | | ★ | ★ | ★ | 9 |
| Wu Y et, 2019 | ★ | ★ | ★ | ★ | ★★ | |  | ★ | ★ | 8 |
| Chen G et, 2013 | ★ | ★ | ★ | ★ | ★ | |  |  | ★ | 6 |
| Huang J et, 2019 | ★ | ★ | ★ | ★ | ★ | |  |  | ★ | 6 |
| Chen H et, 2016 | ★ | ★ | ★ | ★ | ★★ | | ★ |  | ★ | 8 |
| Yang J et, 2015 | ★ | ★ | ★ | ★ | ★★ | |  |  | ★ | 7 |
| Huang Q et, 2024 | ★ | ★ | ★ | ★ | ★ | |  | ★ | ★ | 7 |

**Table 3** **S**ubgroup analysis of RCTs based on TPOAb status

| Study Design | Outcomes | Subgroups | Number of studies | Effect size | Effect P value | *I^2^*（%） |
| --- | --- | --- | --- | --- | --- | --- |
| RCTs | premature birth | TPOAb(±) | 12 | RR=0.89，95%CI=0.46~0.74 | ＜0.0001 | 52 |
|  |  | TPOAb(-) | 6 | RR=0.61，95%CI=0.40~0.92 | 0.02 | 0 |
|  | LBWI | TPOAb(±) | 4 | RR=0.57，95%CI=0.31~1.05 | 0.07 | 58 |
|  |  | TPOAb(-) | 3 | RR=0.54，95%CI=0.26~1.14 | 0.1 | 0 |
|  | macrosomia | TPOAb(-) | 1 | RR=0.29，95%CI=0.06~1.38 | 0.12 | - |
|  | SGA | TPOAb(±) | 1 | RR=1.23，95%CI=0.76~2.00 | 0.40 | - |
|  |  | TPOAb(-) | 1 | RR=0.51，95%CI=0.05~5.58 | 0.58 | - |
|  | birth wieight | TPOAb(±) | 4 | RR=0.14，95%CI=-0.03~0.31 | 0.11 | 95 |
|  |  | TPOAb(-) | 1 | RR=-0.01，95%CI=-0.11~0.09 | 0.84 | - |
|  | TSH in Umbilical cord blood of newborns | TPOAb(±) | 2 | RR=-2.16，95%CI=-4.79~0.48 | 0.11 | 99 |
|  |  | TPOAb(-) | 1 | RR=-3.18，95%CI=-4.25~-2.11 | ＜0.001 | - |
|  | FT3 in Umbilical cord blood of newborns | TPOAb(±) | 2 | RR=0.06，95%CI=-0.24~0.36 | 0.72 | 0 |
|  | FT4 in Umbilical cord blood of newborns | TPOAb(±) | 2 | RR=0.07，95%CI=-0.41~0.56 | 0.77 | 0 |

**Table 4 S**ubgroup analysis of RCTs based on TPOAb status

| Study Design | Outcomes | Subgroups | Number of studies | Effect size | Effect P value | *I^2^*(%) |
| --- | --- | --- | --- | --- | --- | --- |
| cohort studies | premature birth | TPOAb(±) | 9 | RR=0.91，95%CI=0.76~1.09 | 0.32 | 62 |
|  |  | TPOAb(-) | 3 | RR=0.51，95%CI=0.28~0.91 | 0.02 | 0 |
|  | LBWI | TPOAb(±) | 7 | RR=0.71，95%CI=0.57~0.87 | 0.001 | 0 |
|  |  | TPOAb(-) | 2 | RR=0.98，95%CI=0.66~1.44 | 0.91 | 0 |
|  | macrosomia | TPOAb(±) | 2 | RR=1.07，95%CI=0.67~1.72 | 0.78 | 0 |
|  |  | TPOAb(-) | 1 | RR=0.32，95%CI=0.19~0.56 | ＜0.001 | - |
|  | CH | TPOAb(±) | 2 | RR=1.27，95%CI=0.16~10.07 | 0.82 | 0 |
|  | birth wieight | TPOAb(-) | 1 | RR=0.10，95%CI=-0.08~0.28 | 0.28 | - |
|  | TSH in Umbilical cord blood of newborns | TPOAb(-) | 1 | RR=-3.53，95%CI=-4.27~-2.79 | ＜0.001 | - |
|  | FT3 in Umbilical cord blood of newborns | TPOAb(-) | 1 | RR=0.08，95%CI=-0.72~0.88 | 0.84 | - |
|  | FT4 in Umbilical cord blood of newborns | TPOAb(-) | 1 | RR=0.03，95%CI=-1.18~1.24 | 0.96 | - |

# Supplementary material

Search strategy

**Cochrane Library**

#1 MeSH descriptor: [Hypothyroidism] explode all trees 636

#2 (Thyroid Stimulating Hormone Deficiency):ti,ab,kw OR (Thyroid-Stimulating Hormone Deficiencies):ti,ab,kw OR (TSH Deficiencies):ti,ab,kw OR (TSH Deficiency):ti,ab,kw OR (Thyroid-Stimulating Hormone Deficiency):ti,ab,kw 317

#3 (Deficiency, Thyroid-Stimulating Hormone):ti,ab,kw OR (Deficiency, TSH):ti,ab,kw OR (Hormone Deficiency, Thyroid-Stimulating):ti,ab,kw OR (Primary Hypothyroidism):ti,ab,kw OR (Primary Hypothyroidisms):ti,ab,kw 1696

#4 (Hypothyroidism, Primary):ti,ab,kw OR (Hypothyroidisms):ti,ab,kw OR (Hypothyroidism, Central):ti,ab,kw OR (Central Hypothyroidism):ti,ab,kw OR (Hypothyroidism, Secondary):ti,ab,kw 1608

#5 MeSH descriptor: [Pregnancy] explode all trees 34185

#6 ("pregnancies"):ti,ab,kw OR (Gestation):ti,ab,kw OR (pregnancy):ti,ab,kw 84213

#7 #5 OR #6 84560

#8 MeSH descriptor: [Thyroxine] explode all trees 1378

#9 (Thevier):ti,ab,kw OR (Eltroxin):ti,ab,kw OR (Synthroid):ti,ab,kw OR (Unithroid):ti,ab,kw OR (L-Thyroxin Henning):ti,ab,kw 39

#10 (L Thyroxin Henning):ti,ab,kw OR (Levothroid):ti,ab,kw OR (Tiroidine):ti,ab,kw OR (Levothyroid):ti,ab,kw OR (Dexnon):ti,ab,kw 11

#11 (Eltroxine):ti,ab,kw OR (Sodium Levothyroxine):ti,ab,kw OR (Levothyroxine Sodium):ti,ab,kw OR (Eferox):ti,ab,kw OR (Novothyrox):ti,ab,kw 140

#12 (Secondary Hypothyroidism):ti,ab,kw OR (Secondary Hypothyroidisms):ti,ab,kw OR (Central Hypothyroidisms):ti,ab,kw OR (Hypothyroidisms):ti,ab,kw 1023

#13 #1 OR #2 OR #3 OR #4 OR #12 2276

#14 (Levothyroxine):ti,ab,kw OR (L Thyroxine):ti,ab,kw OR (L-Thyroxine):ti,ab,kw OR (L-Thyroxin beta):ti,ab,kw OR (Thyroxine):ti,ab,kw 2765

#15 (L Thyroxin beta):ti,ab,kw OR (Thyrax):ti,ab,kw OR (L Thyroxine Roche):ti,ab,kw OR (L-Thyroxine Roche):ti,ab,kw OR (Levo-T):ti,ab,kw 7

#16 (Levo T):ti,ab,kw OR (Levothyroxin Deladande):ti,ab,kw OR (Levothyroxin Delalande):ti,ab,kw OR (Levoxyl):ti,ab,kw OR (Synthrox):ti,ab,kw 23

#17 (Levoxine):ti,ab,kw OR (L-Thyrox):ti,ab,kw OR (L Thyrox):ti,ab,kw OR (Euthyrox):ti,ab,kw OR (Eutirox):ti,ab,kw 42

#18 (Berlthyrox):ti,ab,kw OR (Tiroxina Leo):ti,ab,kw OR (Novothyral):ti,ab,kw OR (Oroxine):ti,ab,kw OR (Thyroxin):ti,ab,kw 185

#19 (Thyroid Hormone, T4):ti,ab,kw OR (T4 Thyroid Hormone):ti,ab,kw OR (Lévothyrox):ti,ab,kw 735

#20 #8 OR #9 OR #10 OR #11 OR #14 OR #15 OR #16 OR #17 OR #18 OR #19 3237

#21 MeSH descriptor: [Therapeutics] explode all trees 431255

#22 (Therapeutics):ti,ab,kw OR (Therapies):ti,ab,kw OR (Therapeutic):ti,ab,kw OR (Treatments):ti,ab,kw OR (Treatment):ti,ab,kw 1116922

#23 (Therapy):ti,ab,kw 891720

#24 #21 OR #22 OR #23 1392301

#25 (Subclinical):ti,ab,kw OR (Sub-clinical):ti,ab,kw OR (subclin*):ti,ab,kw OR (sub-clin*):ti,ab,kw 4648

#26 #7 AND #13 AND #20 AND #24 AND #25 61

**Pubmed**

("subclinical"[Title/Abstract] OR "sub-clinical"[Title/Abstract] OR "subclin*"[Title/Abstract] OR "sub clin*"[Title/Abstract])

AND (("Pregnancy"[Title/Abstract] OR "Pregnancies"[Title/Abstract] OR "Gestation"[Title/Abstract] OR "Pregnancy"[MeSH Terms])

AND ("Hypothyroidism"[Title/Abstract] OR "Hypothyroidisms"[Title/Abstract] OR "thyroid stimulating hormone deficiency"[Title/Abstract] OR "deficiency thyroid stimulating hormone"[Title/Abstract] OR "hormone deficiency thyroid stimulating"[Title/Abstract] OR "thyroid stimulating hormone deficiencies"[Title/Abstract] OR "thyroid stimulating hormone deficiency"[Title/Abstract] OR "tsh deficiency"[Title/Abstract] OR "deficiency tsh"[Title/Abstract] OR "tsh deficiencies"[Title/Abstract] OR "secondary hypothyroidism"[Title/Abstract] OR "hypothyroidism secondary"[Title/Abstract] OR (("neoplasm metastasis"[MeSH Terms] OR ("neoplasm"[All Fields] AND "metastasis"[All Fields]) OR "neoplasm metastasis"[All Fields] OR "secondaries"[All Fields] OR "Secondary"[MeSH Subheading] OR "Secondary"[All Fields]) AND "Hypothyroidisms"[Title/Abstract]) OR "central hypothyroidism"[Title/Abstract] OR (("Central"[All Fields] OR "centrally"[All Fields] OR "centrals"[All Fields]) AND "Hypothyroidisms"[Title/Abstract]) OR "hypothyroidism central"[Title/Abstract] OR "primary hypothyroidism"[Title/Abstract] OR "hypothyroidism primary"[Title/Abstract] OR "primary hypothyroidisms"[Title/Abstract] OR "Hypothyroidism"[MeSH Terms]) AND

("3 5 3 5 tetraiodothyronine"[Title/Abstract] OR "t4 thyroid hormone"[Title/Abstract] OR "thyroid hormone t4"[Title/Abstract] OR "Thyroxin"[Title/Abstract] OR (("o"[All Fields] AND "4 hydroxy 3 5 diiodophenyl"[All Fields]) AND "3 5 diiodotyrosine"[Title/Abstract]) OR "Eltroxine"[Title/Abstract] OR "Novothyral"[Title/Abstract] OR "Levothyroid"[Title/Abstract] OR "levothyroxine sodium"[Title/Abstract] OR "sodium levothyroxine"[Title/Abstract] OR "Levoxine"[Title/Abstract] OR "Synthroid"[Title/Abstract] OR "Levoxyl"[Title/Abstract] OR "Dexnon"[Title/Abstract] OR "Eferox"[Title/Abstract] OR "Eltroxin"[Title/Abstract] OR "Euthyrox"[Title/Abstract] OR "Eutirox"[Title/Abstract] OR ("L-Thyroxin"[All Fields] AND "beta"[Title/Abstract]) OR (("L"[All Fields] AND ("Thyroxine"[MeSH Terms] OR "Thyroxine"[All Fields] OR "Thyroxin"[All Fields] OR "thyroxines"[All Fields])) AND "beta"[Title/Abstract]) OR "l thyroxin henning"[Title/Abstract] OR "l thyroxin henning"[Title/Abstract] OR (("Thyroxine"[MeSH Terms] OR "Thyroxine"[All Fields] OR "L-Thyroxine"[All Fields]) AND "Roche"[Title/Abstract]) OR (("Thyroxine"[MeSH Terms] OR "Thyroxine"[All Fields] OR "L-Thyroxine"[All Fields]) AND "Roche"[Title/Abstract]) OR ("L"[All Fields] AND "Thyrox"[Title/Abstract]) OR "Levo-T"[Title/Abstract] OR "Levo-T"[Title/Abstract] OR "Levothroid"[Title/Abstract] OR (("laevothyroxine"[All Fields] OR "Thyroxine"[MeSH Terms] OR "Thyroxine"[All Fields] OR "Levothyroxine"[All Fields] OR "Levothyroxin"[All Fields]) AND "Delalande"[Title/Abstract]) OR "Thyrax"[Title/Abstract] OR "Levothyrox"[Title/Abstract] OR ("Tiroxina"[All Fields] AND "Leo"[Title/Abstract]) OR "Unithroid"[Title/Abstract] OR "Levothyroxine"[Title/Abstract] OR "l 3 5 3 5 tetraiodothyronine"[Title/Abstract] OR "L-Thyroxine"[Title/Abstract] OR "L-Thyroxine"[Title/Abstract] OR (("o"[All Fields] AND "4 hydroxy 3 5 diiodophenyl"[All Fields]) AND "3 5 diiodo l tyrosine"[Title/Abstract]) OR "Thyroxine"[Title/Abstract] OR "Thyroxine"[MeSH Terms]) AND

("Therapeutics"[MeSH Terms] OR ("Therapeutics"[Title/Abstract] OR "Therapeutic"[Title/Abstract] OR "Therapy"[Title/Abstract] OR "Therapies"[Title/Abstract] OR "Treatment"[Title/Abstract] OR "Treatments"[Title/Abstract])))
